# Plant Genetics and Gene Study

**DOI:** 10.1155/2019/3560374

**Published:** 2019-04-04

**Authors:** Yuri Shavrukov, Nikolai Borisjuk, Narendra K. Gupta

**Affiliations:** ^1^Flinders University, College of Science and Engineering, Biological Sciences, Adelaide, Australia; ^2^School of Life Science, Huaiyin Normal University, Huaian, China; ^3^Rajasthan Agricultural Research Institute, SKN Agriculture University, Durgapura, Jaipur, India

Plants are the primary source of human food and animal feed and also form the basis of numerous industrial and pharmaceutical products. This special issue reflects the diversity of modern research in the field of plant molecular genetics. It covers a wide range of modern technologies and scientific approaches that aim to achieve a better understanding of the various aspects of molecular mechanisms underpinning the key traits in major crops and other commercially important plant species.

Three papers in this issue deal with the development, study, and application of molecular markers in plant breeding. N. Kim et al. in “Development of Clustered Resistance Gene Analogs-Based Markers of Resistance to* Phytophthora capsici* in Chili Pepper” reported on 11 novel molecular markers targeting resistance to the soil-borne pathogen* Phytophthora capsici* in chili pepper. The markers developed through high-resolution melting analysis, represent an excellent tool for marker-assisted selection. Another type of molecular markers, SSR, was used for the study of genetic diversity in cultivated and wild melon (J. Hu et al. “Microsatellite Markers Reveal Genetic Diversity and Relationships within a Melon Collection Mainly Comprising Asian Cultivated and Wild Germplasms”). The authors found enormous genetic variability within the collection, and deployment of the well-known SSR markers enabled the generation of a phylogenetic tree showing the relationships between melon accessions. An investigation presented by R. Ben Ayed and A. Rebai (“Tunisian Table Olive Oil Traceability and Quality Using SNP Genotyping and Bioinformatics Tools”) revealed a significant link between the five SNP markers analysed and the biochemical composition and quality of olive fruits.

Several of the presented reports are related to the genetic control of plant response to abiotic stresses. Drought is one of the major challenges being faced in agriculture. The paper presented by N. M. Kamal et al. (“Stay-Green QTLs Response in Adaptation to Post-Flowering Drought Depends on the Drought Severity”) provides important findings on QTLs identified in sorghum grown in Sudan (Africa) under drought conditions. The presented data, generated in cooperation with the International Atomic Energy Agency (IAEA), Austria, can be used for the improvement of grain yield in drought-prone environments using a “stay-green” approach through the application of QTL analysis. Tolerance to aluminum toxicity represents an entirely different type of abiotic stress, but this too is an important agricultural problem, especially in acidic soils. The paper “Aluminum Responsive Genes in Flax (*Linum usitatissimum* L.)” presented by G. S. Krasnov et al. reported valuable results from RNAseq analysis and candidate gene identification for flax genotypes tolerant or sensitive to a high concentration of Al. The authors' conclusions on glutathione metabolism, oxidoreductase, and transmembrane transporters can be further applied both in academic study and in practical breeding. Very few investigations have been conducted on plant growth in the absence of gravity during space-shuttle orbit around our planet. The paper presented by O. Yu. Yurkevich et al. (“Molecular Cytogenetics of* Pisum sativum* L. Grown under Spaceflight-Related Stress”) describes a chromosome analysis of pea progenies derived from plants grown in space using a novel FISH approach. Minor chromosome rearrangements were observed in response to “spaceflight-related stress,” which could lead to better guidelines for biological experiments with plants during future space-shuttle missions.

IAEA also supported a study on the generation of mutant bread wheat with improved micronutrient content in the grain. S. Kenzhebayeva et al. presented their paper “Mutant Lines of Spring Wheat with Increased Iron, Zinc, and Micronutrients in Grains and Enhanced Bioavailability for Human Health,” where stable breeding lines of wheat were produced from the M_7_ generation. The authors reported a two-fold increase of Fe, Zn, and Ca, and reduced levels of the anti-nutrient phytic acid in grains, which can be directly used in practical wheat breeding. In addition to radiation or chemical mutagenesis, inherited changes could be induced by “genomic shock” in plants generated through interspecies hybridization. This phenomenon was studied in rye-wheat hybrids named “secalotriticum” by Y. A. Lipikhina et al. in their paper “Dynamics of the Centromeric Histone* CENH3* Structure in Rye-Wheat Amphidiploids (Secalotriticum).” This is an extremely intricate cytogenetic study of the centromeric nucleosomes in dividing cells. The authors presented some critical results from* CENH3* gene analysis, a central player in this very complex cytological trait, offering promising potential outcomes.

A remarkable level of genetic polymorphism was found in a germplasm collection of* Phaseolus *beans using diverse characteristics derived from the seed morphology of these species. L. Sinkovič et al. presented their paper “Morphological Seed Characterization of Common (*Phaseolus vulgaris* L.) and Runner (*Phaseolus coccineus* L.) Bean Germplasm: A Slovenian Gene Bank Example,” which contained an interesting comparison of seeds, accompanied by color images. The presented findings are essential for a better understanding of the wide diversity of bean species, which can be directly used for genetics and breeding of these nutritionally important crop varieties. In contrast, another paper “*In Silico* Genome-Wide Analysis of the ATP-Binding Cassette Transporter Gene Family in Soybean (*Glycine max* L.) and Their Expression Profiling,” presented by A. K. Mishra et al., reported on computer analyses of available databases concerning the important gene family of the* ATP-binding cassette transporter*, in soybean. The authors provided a very accurate and detailed analysis of all identified genes and protein isoforms, arranging these new findings within the context of other pertinent information and comparisons.

P. Soundararajan et al. presented a detailed review entitled “Insight on Rosaceae Family with Genome Sequencing and Functional Genomics Perspective.” The Rosaceae family is one of the most significant plant families, and the fruits, berries, flowers, and many other important parts of these plants are familiar to each of us from early childhood. The authors have provided a comprehensive overview on the modern level of genetic knowledge and technology employed in comparative genomics and functional genome analysis for many Rosaceae species. Whole genome sequences in this plant family are so important for genetics, breeding, and agriculture that the presented review will surely be of broad general interest to future investigations in this area.

In conclusion, the review presented by N. Borisjuk et al., entitled “Genetic Modification for Wheat Improvement: From Transgenesis to Genome Editing,” summarizes the attempts and results of wheat improvement using various genetic engineering approaches. The authors reflect the progress achieved since the dawn of genetic transformation up until the recent emergence of precise modern genome editing using CRISPR/Cas9 and related systems in wheat. This comprehensive review can provide a strong stimulus for both current and future researchers to pursue work on wheat improvement, and deploy all available modern technologies to secure a sustainable supply of quality agricultural produce for our growing global population.

*Yuri Shavrukov* *Nikolai Borisjuk* *Narendra K. Gupta*

